# High Microsatellite but No Mitochondrial DNA Variation in an Invasive Japanese Mainland Population of the Parasitoid Wasp *Melittobia sosui*

**DOI:** 10.1002/ece3.71026

**Published:** 2025-02-15

**Authors:** Jun Abe, Jun‐ichi Takahashi, Koji Tsuchida

**Affiliations:** ^1^ Faculty of Sciences Kanagawa University Kanagawa Japan; ^2^ Faculty of Life Sciences Kyoto Sangyo University Kyoto Japan; ^3^ Faculty of Applied Biological Sciences Gifu University Gifu Japan

**Keywords:** biological invasion, climate change, development of microsatellite DNA markers, dispersal, population genetic analysis, *Wolbachia*‐induced sweep

## Abstract

Invasive populations are predicted to have reduced genetic diversity due to bottleneck events. The parasitoid wasp *Melittobia sosui* was previously identified only in the subtropical area of the southern Japanese islands and Taiwan but was recently found in the temperate area of the Japanese mainland. The distribution of this species may have recently expanded northward due to factors such as climatic events and global warming. The population genetics of both the native and invasive regions were investigated using mitochondrial and nuclear microsatellite DNA. As expected, mitochondrial variation was observed in the native region but not in the invasive region, which had only one haplotype. However, the two regions exhibited similar levels of microsatellite variation, and an average of 43% and 38% of alleles were uniquely found in the native and invasive populations, respectively. The difference in genetic variation between mitochondrial and microsatellite DNA in the invasive populations may be explained by the faster mutation rate of microsatellites, as well as the population structure of *Melittobia*, in which the subdivision into small inbreeding lineages may facilitate the accumulation of mutations. The high proportion of private alleles suggests that the mainland population diverged from the native populations at least 100 years ago, ruling out the possibility that the mainland population was established recently. The present study suggests that *M. sosui* might have already existed on the mainland but at a low frequency or that the mainland population was derived from a ghost population that diverged from the native populations more than 100 years ago.

## Graphical abstract

The parasitoid wasp *Melittobia sosui* was originally distributed only in the subtropical regions of Japan's Ryukyu Islands and Taiwan, but it has recently been found in the temperate regions of the Japanese mainland as well. This study examined the population genetic structure and genetic diversity between native and invasive populations and found reduced variation in mitochondrial DNA but relatively high variation in nuclear DNA in the invasive mainland population.

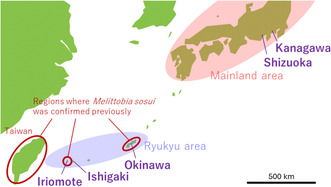

## 1 Introduction

How populations of species are established in novel habitat areas outside the typical distribution range of the species is a fundamental question in evolutionary ecology and conservation biology. The distributions of species are changing at accelerating rates due to natural processes as well as human activities (Pecl et al. 2017). Organisms naturally expand their distribution through climatic events such as typhoons and hurricanes. Additionally, human transport has facilitated the expansion of organisms beyond their natural migration ranges (Hulme 2009; Banks et al. 2015). Recent climate changes, such as global warming, have led to the expansion of distribution ranges, mainly toward higher latitudes, in a wide range of organisms (Deutsch et al. 2008; Walther et al. 2009).

Through the process of invasion, the genetic diversity of invasive populations can change from that of native populations. Owing to bottleneck events, through which only a fraction of genetic variants are transferred from native habitats by founders, invasive populations often exhibit lower genetic diversity than native populations do (Nei et al. 1975). However, multiple introductions or introductions by numerous founders can prevent a reduction in genetic diversity or even maintain genetic diversity at a level comparable to that of the native population (Dlugosch and Parker 2008; Uller and Leimu 2011). Along with variation introduced by founders, variants can arise from mutations after divergence (Talla et al. 2019; Kaňuch et al. 2021). In addition, infection by the endosymbiotic bacteria such as *Wolbachia*, which infect a wide range of arthropods and nematodes, has frequently been reported to affect the genetic structure and diversity of their host species (Werren et al. 2008). *Wolbachia* are maternally transmitted and often manipulate the reproduction of their hosts in various ways, such as parthenogenesis, feminization, male‐killing, and cytoplasmic incompatibility (Hurst 1993; Werren 1997; Stouthamer et al. 1999). These reproductive manipulations enhance the vertical transmission of *Wolbachia* lineages, coupled with maternally co‐inherited mitochondrial haplotypes. Therefore, *Wolbachia*‐induced sweep causes a reduction in mitochondrial variation but has little effect on nuclear variation (Hale and Hoffmann 1990; Werren et al. 2008; Raychoudhury et al. 2010; Miyata et al. 2017).

**FIGURE 1:**
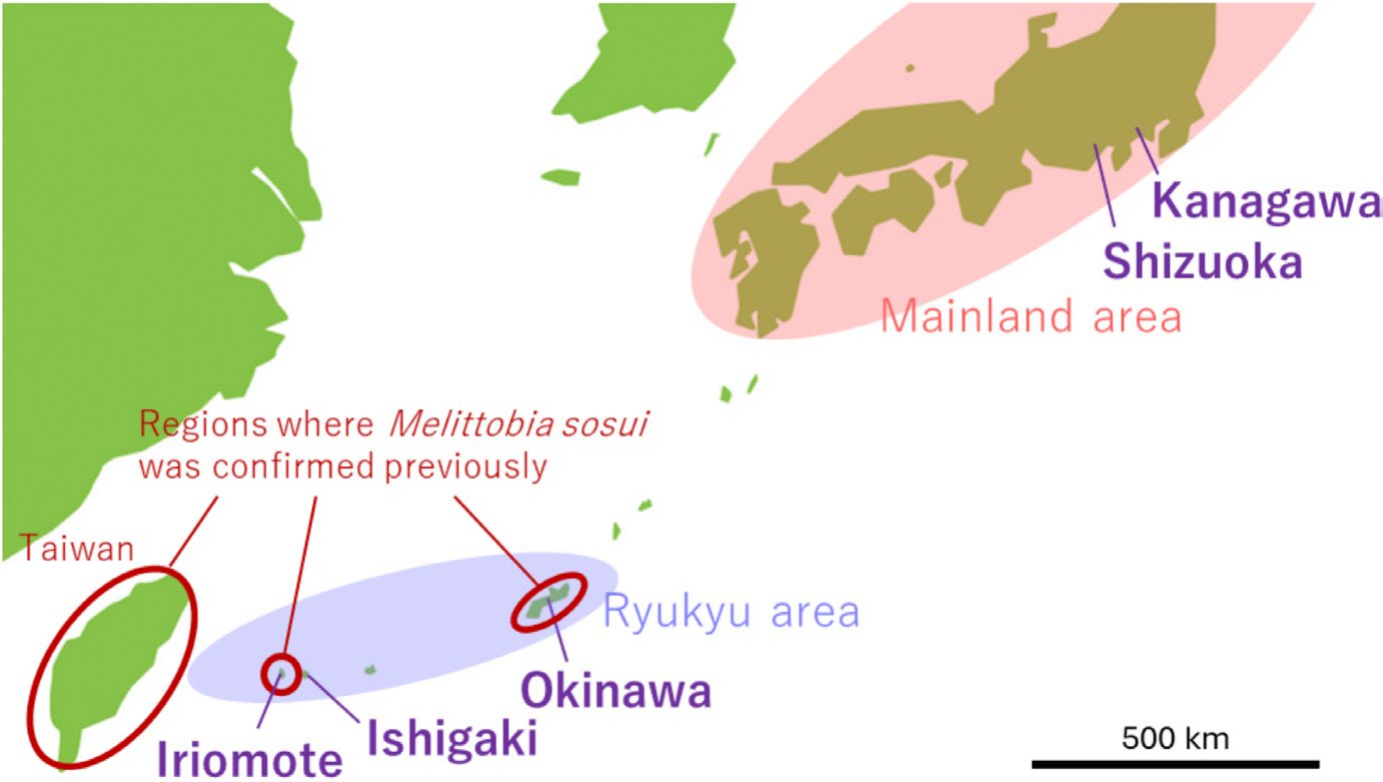
Map of the Japanese mainland and Ryukyu areas and Taiwan. *Melittobia sosui* was previously reported in Okinawa, Iriomote, and Taiwan. The present study sampled *M. sosui* from the native areas of Iriomote, Ishigaki, and Okinawa in the Ryukyu area as well as from the new habitats of Shizuoka and Kanagawa in the mainland area.

*Melittobia sosui* is a parasitoid wasp that attacks the larvae and pupae, mainly prepupae, of a wide range of solitary wasp and bee species nesting above ground (Matthews et al. 2009). Although the distribution of this species has not necessarily been extensively studied, *M. sosui* has been confirmed to inhabit only in the subtropical regions of the southern Japanese islands (Okinawa and Iriomote islands) and Taiwan, but it has not been found in the temperate regions of the Japanese mainland (Figure 1; Assem and Maeta 1980; Assem et al. 1982; Maeta 1985; Maeta et al. 1996). However, *M. sosui* has been identified in Shizuoka and Kanagawa in the Japanese mainland since 2008 (Figure 1; Jun Abe unpublished data).

The reproduction of *Melittobia* species is characterized by repeated inbreeding, as mating occurs between individuals that developed on the same host, and related females that did not disperse from their natal patch often lay eggs together on a host (Matthews et al. 2009; Abe and Pannebakker 2017; Abe et al. 2021). *Melittobia* is haplodiploid, like other Hymenopteran species. Deleterious mutations are hidden in diploid females but purged by selection in haploid males, leading to less reduction in fitness through inbreeding depression (Werren 1993). Moreover, unlike many other Hymenopteran species, chalcid wasps including *Melittobia* have a sex determination system other than complementary sex determination (CSD); therefore, inbreeding does not cause genetic load by producing sterile diploid males (Beukeboom and Perrin 2014). Therefore, even if only a single mated female invades a new habitat area and conditions in the environment are suitable, she is likely to establish a new population that does not suffer from inbreeding depression. Because the host species build their nests in the gaps and crevices of not only natural but also artificial structures, hosts parasitized by *M. sosui* may immigrate via human transports. Additionally, a jet stream travels from the southwest to the northwest in the area. Recent global warming has been reported to have caused the northward expansion of various insect species from southern regions of the Japanese archipelago (Kiritani 2006). Therefore, the most straightforward and plausible scenario could be that the newly identified *M. sosui* population in the Japanese mainland has been established relatively recently (since the 1980s) by an immigrant(s) from the southwest regions.

In this study, we examined the genetic structure and variation of *M. sosui* at the levels of nuclear and mitochondrial DNA in both the native region (Ryukyu) and the new habitat region (mainland area; Figure 1). We expected reduced genetic diversity in both the nuclear and mitochondrial DNA of the populations from the new habitat, if gene flow from the native area had occurred recently. Most nuclear microsatellite DNA markers identified for other *Melittobia* species do not apply to the present species; therefore, we developed new microsatellite markers for *M. sosui* using next‐generation sequencing (Abe and Pannebakker 2017). Moreover, because *Wolbachia* might affect the genetic structure and diversity of the populations, we also investigated *Wolbachia* infection in the *M. sosui* populations. Finally, based on the results, we determined how *M. sosui* was introduced and settled in the Japanese mainland and the consequent changes in genetic diversity during the subsequent population expansion.

## 2 Materials and Methods

### 2.1 Development of Microsatellite DNA Markers

DNA was extracted from 20 *M. sosui* individuals of a laboratory strain originating from individuals collected at Hiratsuka, Kanagawa, in 2017. Whole bodies were homogenized and digested in 500 μL of 10 mM Tris–HCl (pH 8.0) supplemented with 10 mM EDTA, 0.5% SDS, and 0.5 mg/mL proteinase K at 55°C for 24 h. Genomic DNA was extracted from the solution once with phenol, once with phenol/chloroform (1:1), and once with chloroform/isoamyl alcohol (24:1). DNA was then precipitated with ethanol/3 M sodium acetate (20:1), washed with 70% ethanol, dried, and dissolved in TE buffer. The DNA samples were stored at −20°C until further use.

Microsatellite DNA motifs were sequenced with Illumina's MiSeq platform (Illumina). DNA quantification of the solutions was conducted with Synergy LX (BioTek) and the QuantiFluor dsDNA System (Promega). DNA fragmentation was conducted using a Covaris device under conditions that resulted in the generation of DNA fragments with a length of 500 base pairs. Library preparation was conducted in accordance with the manufacturer's instructions, utilizing sheared DNA (50 ng) and the KAPA Hyper Prep Kit. The libraries were subjected to 8 cycles of polymerase chain reaction (PCR) amplification. Adapters from the FastGene Adapter Kit (FastGene) were used. Quantification of the libraries was conducted using Synergy H1 and the QuantiFluor dsDNA System to determine the concentrations of the prepared libraries. The quality of the libraries was evaluated using a Bioanalyzer and a high Sensitivity DNA kit (Agilent Technologies). Sequencing analysis was performed on the MiSeq platform under 2 × 300 bp conditions using the prepared libraries. The raw sequence data were stripped of adapter sequences and low‐quality bases using default parameters. The data were adjusted through quality filtering using Sickle (Joshi and Fass 2011). Bases with a quality value below 20 were removed, and paired reads were discarded if they had fewer than 120 bases. Clean reads were obtained by assembling high‐quality reads using Spades ver 3.10.1.

The reads from the raw sequence data containing microsatellite regions were screened using the MSATCOMMANDER program (Faircloth 2008). The selection criteria for microsatellite motifs were at least 15 consecutive complete dinucleotide repeat sequences or at least 10 consecutive complete trinucleotide repeat sequences. The microsatellite primers used were designed to be at least 20 bases away from the microsatellite region and at least 100 bp when amplified by PCR. Microsatellite primers were designed using Primer3Plus software (Untergasser et al. 2007) under these criteria. To assess the amplification and polymorphism for each primer pair designed, PCR was conducted using the M13‐tail technique (Schuelke 2000), with a total volume of 5 μL, containing 2.5 μL of 2× Type‐it multiplex PCR Master Mix (QIAGEN), 0.5 μL of M13 primer (10 μM) labeled with a fluorescent dye (6‐FAM, VIC, NED, or PET), 0.5 μL of 10× primer mixture (0.05 μM forward primer with M13 tail and 0.2 μM reverse primer), and 1.5 μL of genomic DNA. The temperature profile was as follows: 5 min at 94°C; 30 cycles of 30 s at 94°C, 45 s at 60°C, and 45 s at 72°C; 8 cycles of 30 s at 94°C, 45 s at 53°C, and 45 s at 72°C; and a final extension of 10 min at 72°C. The PCR products were electrophoresed with an ABI 3130 sequencer (Thermo Fisher) and analyzed with Peak Scanner software version 1.0 (Thermo Fisher).

Tests for linkage disequilibrium between each locus pair were performed with the software GENEPOP version 4.7 (Rousset 2008), and significance thresholds were corrected using the false discovery rate (FDR; Benjamini and Hochberg 1995). As high genetic differentiation was observed between the Ryukyu and mainland population groups (as shown below), the linkage disequilibrium test was performed separately for each population group.

### 2.2 Sample Collection and DNA Extraction

We conducted field work in the native Ryukyu area, which comprised the Iriomote, Ishigaki, and Okinawa populations, and the invasive mainland area, which comprised the Shizuoka and Kanagawa populations (Figure 1). We set bamboo traps on Iriomote island in 2008–2015; Ishigaki island in 2008–2015 and 2021–2023; Okinawa island in 2008–2009; Fukuroi city, Shizuoka in 2008; and in Hiratsuka city, Kanagawa in 2011–2023 (Abe and Pannebakker 2017; Abe et al. 2021). The bamboo canes in which solitary wasps and bees nested were brought back to the laboratory and inspected for parasitism by *Melittobia* species. *Melittobia* juveniles were incubated at 25°C until emergence. After the species were identified, the emerged individuals were preserved in 99.5% ethanol.

Total genomic DNA was extracted using a DNeasy Blood & Tissue Kit (Qiagen) according to the manufacturer's protocol. We examined 6, 10, and 7 individuals from the Iriomote, Ishigaki, and Kanagawa populations, respectively, to determine the polymorphisms of the identified microsatellite markers (Tables S1–S9). For the mitochondrial and microsatellite population genetics analyses, we added additional samples and examined 14, 25, 3, 1, and 10 individuals from the Iriomote, Ishigaki, Okinawa, Shizuoka, and Kanagawa populations, respectively (Tables S1–S9). We sampled only one individual per bamboo trap to avoid collecting related individuals (Abe et al. 2021). All the individuals were female except one from the Okinawa population, as only several male individuals emerged from the brood. As *M. sosui* is haplodiploid and males are haploid, we calculated population genetic estimates based on heterozygote frequencies, such as *F*_IS_ and *F*_ST_, using only female individuals and discarding the male individual.

### 2.3 | Mitochondrial DNA Analysis

PCR was performed to amplify the mitochondrial cytochrome oxidase subunit I (COI) gene region using the primer pair LCO1490 (5′‐GGTCAACAAATCATAAAGATATTGG‐3′) and HCO2198 (5′‐TAAACTTCAGGGTGACCAAAAAATCA‐3′) (Folmer et al. 1994). PCR was conducted with an ABI 2720 thermal cycler (Thermo Fisher) in a total volume of 10 μL, containing 1 μL of Ex Taq buffer, 0.8 μL of dNTP mixture (2.5 μM each), 0.1 μL of Ex Taq Hot Start Version (TaKaRa Bio), 2.5 μL of each primer (2 μM), and 1 μL of genomic DNA. The temperature profile was as follows: 5 min at 96°C; 30 cycles of 30 s at 94°C, 45 s at 50°C, and 45 s at 68°C; and a final extension of 2 min at 72°C. After the PCR products were treated with ExoSAP‐IT Express (Thermo Fisher), sequencing was conducted for both strands with the same primers used for the PCR, using a BigDye Terminator v3.1 Cycle Sequencing Kit (Thermo Fisher). The sequencing products were purified with a gel filtration cartridge (Edge BioSystems), and the products were analyzed using an ABI 3130 sequencer (Thermo Fisher).

Both strands were assembled using GeneStudio Professional, version 2.2.0.0 (GeneStudio Inc.), and multiple sequence alignments were performed with the ClustalW program (Thompson et al. 2003) implemented in MEGA11 version 11.0.13 (Tamura et al. 2021). Sequences of three nucleotides that could not be determined unambiguously were excluded, and the remaining 532 bp were used for subsequent analyses. Using the sequence data, a haplotype network was constructed using the TCS network implemented in PopART version 1.7 (Leigh and Bryant 2015). To investigate the level of mitochondrial diversity, the number of haplotypes, haplotype diversity, and nucleotide diversity were calculated using DnaSP version 6.12.03 (Rozas et al. 2017).

### 2.4 | Microsatellite DNA Analysis

PCR was conducted under the same conditions as those used for the amplification and polymorphism checks above using primer pairs developed for microsatellite loci. The observed and expected heterozygosities (*H*_O_ and *H*_E_), the inbreeding coefficient (*F*_IS_) according to Weir and Cockerham (1984), and the deficiency of Hardy–Weinberg equilibrium (HWE) were calculated using GENEPOP version 4.7 (Rousset 2008) and Arlequin version 3.5.2.2 (Excoffier and Lischer 2010). Pairwise *F*_ST_ values between populations were estimated using Arlequin. To investigate genetic differentiation between the native Ryukyu group and the invasive mainland group, analysis of molecular variation (AMOVA) was carried out using Arlequin.

To estimate the population genetic structure across all the regions, Bayesian clustering analysis was performed using STRUCTURE version 2.3.4 (Pritchard et al. 2000). For the admixture model, the predefined number of genetic clusters to which individuals were assigned (*K*) was set from one to six, and 10 independent runs were repeated for each value of *K*. Each run involved 1,000,000 Markov chain Monte Carlo iterations after a burn‐in period of 100,000 iterations. The most likely value of *K* was selected based on the Δ*K* method (Evanno et al. 2005) using STRUCTURE HARVESTER (Earl and Holdt 2012). The runs of the most likely *K* value were summarized using CLUMPAK (Kopelman et al. 2015). To investigate the population structure across the regions, phylogenetic trees were also constructed with the neighbor‐joining (NJ) method (Saitou and Nei 1987) using Nei's *D*_A_ genetic distance in POPTREE2 (Takezaki et al. 2010), and graphically edited using MEGA11.

The genetic diversity indices, specifically the number of alleles, gene diversity, and allelic richness, were calculated for each population and for the Ryukyu and mainland population groups using FSTAT version 2.9.4 (Goudet 1995). The number and frequency of shared and private alleles between the Ryukyu and mainland groups were manually calculated from the number of alleles for each locus.

### 2.5 | PCR Detection for *Wolbachia* Infection

To investigate *Wolbachia* infection, diagnostic PCR was performed to amplify the *Wolbachia* surface protein (*wsp*) gene region using the universal primer set wspF (5′‐GGGTCCAATAAGTGATGAAGAAAC‐3′) and wspR (5′‐TTAAAACGCTACTCCAGCTTCTGC‐3′) (Kondo et al. 2002; Abe et al. 2003). The composition of the reaction mixture was the same as that for mitochondrial COI above, and the temperature profile was as follows: 2 min at 94°C, followed by 30 cycles of 1 min at 94°C, 1 min at 50°C, and 1 min at 70°C. As a positive control, the same PCR was conducted with an individual from a laboratory strain of *Melittobia australica* that was confirmed to be infected by *Wolbachia*. The PCR products were electrophoresed with agarose gels, and the amplification of approximately 0.6 kb segments was checked.

## 3 Results

### 3.1 Development of Microsatellite DNA Markers

A total of 1,341,634 raw reads were obtained from an *M. sosui* gDNA library by Illumina paired‐end sequencing. Microsatellite markers were successfully designed for 32,315 and 1927 primer pairs that amplified the microsatellite regions of dinucleotide and trinucleotide repeats, respectively. We selected 112 primer pairs for trinucleotide repeats to avoid overlap of alleles due to stutter artifacts in dinucleotide repeats. Among those, 101 loci were successfully amplified, and 65 loci had polymorphisms. Among the loci with polymorphism, 55 were selected for subsequent analyses (Table S2). Tests for linkage disequilibrium revealed that there was no linkage disequilibrium in the Ryukyu group, whereas two pairs of loci (Mso007–Mso024 and Mso024–Mso045) in the mainland group were significantly associated with each other (Table S3). As the P value of the other pair of loci (Mso007–Mso045) was also small and fairly close to the corrected significance threshold, we discarded the two loci Mso024 and Mso045, and used the remaining 53 loci for further analyses (Table S2; the sequence data of the microsatellite loci were deposited in DDBJ/ENA/GenBank under the accession number LC848221–LC848279).

### 3.2 Mitochondrial DNA Analysis

**FIGURE 2:**
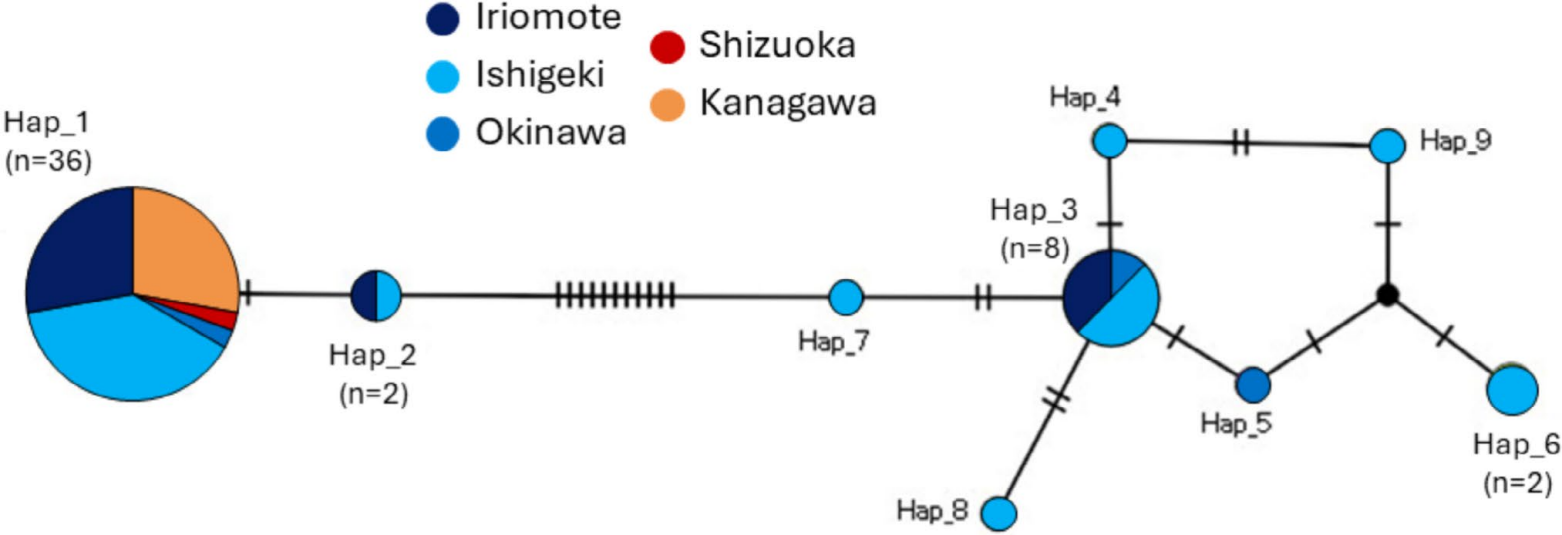
TSC haplotype network of *Melittobia sosui* based on mitochondrial COI sequences. The size of the circles is proportional to the number of individuals sampled: Dark blue, Iriomote; light blue, Ishigaki; blue, Okinawa; red, Shizuoka; orange, Kanagawa. The number of crossbars on the branches represents the number of mutational changes between haplotypes.

In total, we found nine unique mitochondrial haplotypes containing 18 polymorphic sites (the sequence data of all individuals analyzed were deposited in DDBJ/ENA/GenBank under the accession number LC84064–LC848116). The haplotypes were divided into two discrete clades with at least 11 nucleotide differences (Figure 2). The individuals of the populations from the Ryukyu group were distributed into both clades, while all individuals from the mainland populations shared the same haplotype. The haplotype of mainland individuals (Hap1 in Figure 2) was the most common haplotype shared with individuals from the Ryukyu populations. Haplotype diversity and nucleotide diversity in the Ryukyu group were positive, indicating that there was variation in mitochondrial DNA, but the mainland group had no variation (Table 1).

**TABLE 1:** Genetic diversity indices of mitochondrial COI haplotypes in *Melittobia sosui*.

Population^a^	Number of individuals analyzed	Number of haplotypes	Haplotype (gene) diversity	Nucleotide diversity
Per population				
Iriomote	14	3	0.473	0.0097
Ishigaki	25	8	0.673	0.0137
Kanagawa	10	1	0	0
Per group^b^				
Ryukyu	42	9	0.617	0.0127
Mainland	11	1	0	0

^a^ Only populations with sufficient sample sizes are represented.

^b^ The Ryukyu group consists of the Iriomote, Ishigaki, and Okinawa populations, and the mainland group consists of the Shizuoka and Kanagawa populations.

### 3.3 Microsatellite DNA Analysis

The observed heterozygosity *H*_O_, expected heterozygosity *H*_E_, and inbreeding coefficient *F*_IS_ were calculated for loci with polymorphisms and for populations with sufficient sample sizes. Overall, the inbreeding coefficient *F*_IS_ was high, and 6 out of 12, 10 out of 12, and 5 out of 15 loci in the Iriomote, Ishigaki, and Kanagawa populations, respectively, exhibited significant departure from HWE (Table S4). The *F*_IS_ values calculated over all the loci for these populations were 0.816, 0.798, and 0.557, respectively, and significant departure from HWE was detected for all the populations (all *p* < 0.001). This result is reasonable, given the life history of *Melittobia* with repeated inbreeding (Matthews et al. 2009; Abe and Pannebakker 2017; Abe et al. 2021). All the estimates of pairwise *F*_ST_ between the populations were also high and significantly deviated from zero, although the pairwise *F*_ST_ values were relatively higher between the native and invasive populations than within the native populations (Table S5). The AMOVA results, which placed the Ryukyu and mainland populations in separate groups, also revealed that although all three hierarchy levels were significant (all *p* < 0.001), the majority of the variation occurred among groups (*F*_CT_ = 0.871; Table S6), indicating that there was a strong genetic differentiation between the native and invasive populations.

**FIGURE 3:**
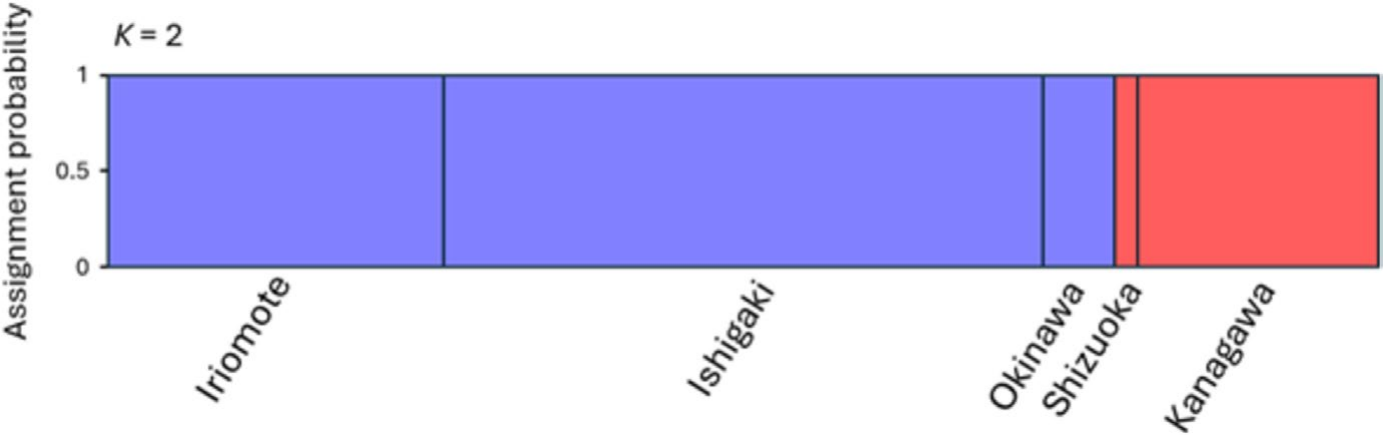
Population genetic structure of five populations of *Melittobia sosui*, as estimated by Bayesian clustering analysis with STRUCTURE based on 53 polymorphic microsatellite DNA markers. The inferred assignment probability that individuals belong to each genetic cluster was determined using the most likely number of clusters according to the Δ*K* method (*K* = 2).

**FIGURE 4:**
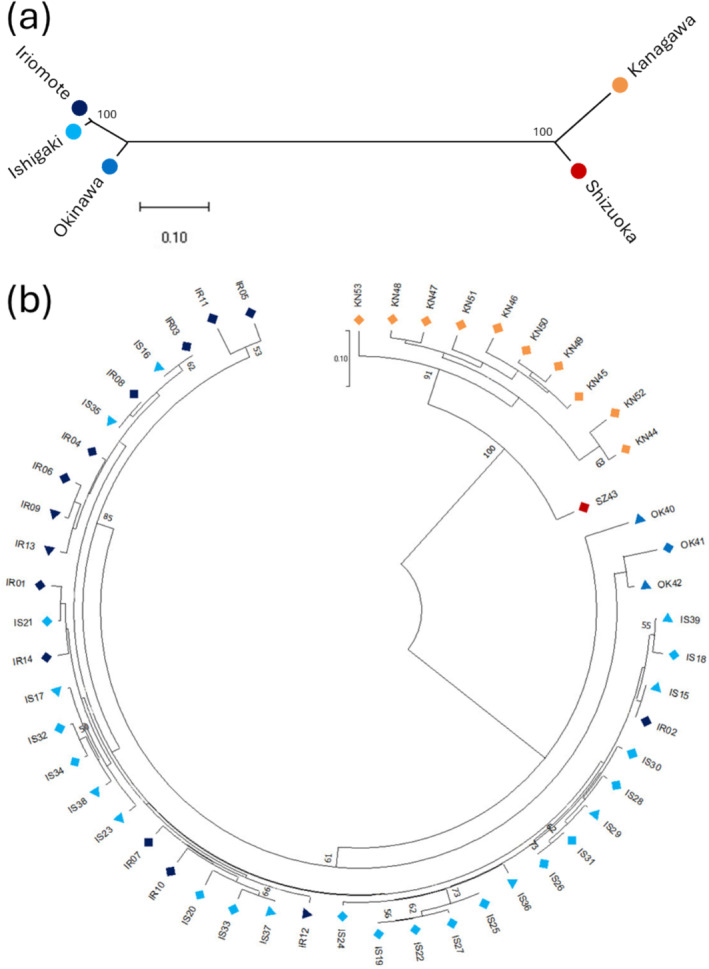
A population‐level neighbor‐joining tree (a) and an individual‐level neighbor‐joining tree (b) of *Melittobia sosui* from the native (Iriomote, Ishigaki, and Okinawa) and invasive (Shizuoka and Kanagawa) populations, based on 53 polymorphic microsatellite DNA markers. The colors of the symbols indicate the population sampled: Dark blue, Iriomote (IR); light blue, Ishigaki (IS); blue, Okinawa (OK); red, Shizuoka (SZ); orange, Kanagawa (KN). The shape of the symbols in b indicates the mitochondrial haplotype clades: Diamond, left clade; triangle, right clade shown in Figure 2. The numbers at each node indicate bootstrap values (only ≥ 50% values are shown). The scale bar indicates genetic distance.

The STRUCTURE analysis revealed that the most likely number of genetic clusters across the entire sampled area was *K* = 2 (Table S7). The two genetic clusters corresponded exactly to the Ryukyu and mainland populations, with all individuals assigned to the expected cluster with almost 100% probability (Figure 3). The NJ tree revealed the same trend, exhibiting two discrete clades containing the Ryukyu and mainland populations with long distances between the clades (Figure 4). Although the mitochondrial haplotypes were also divided into two separate clades (Figure 2), these clades were independent of the phylogeny constructed with microsatellite data. In the individual‐level NJ tree, the mitochondrial haplotype clades did not form monophyletic groups or show any signs of divergence (Figure 4b). These findings suggested that genetic variation in mitochondrial and nuclear microsatellite DNA were not correlated with each other.

The genetic diversity indices calculated using microsatellite loci were within the same range and not significantly different between the Ryukyu and mainland populations (Table 2 and Table S8; Wilcoxon signed rank test: number of alleles, *p* = 0.21; allelic richness, *p* = 0.89; gene diversity, *p* = 0.70). Among 101 microsatellite loci that were successfully amplified, 41 and 46 (40.6% and 45.5%) loci had private alleles that were present only in the Ryukyu and mainland populations, respectively. On average, over the 53 loci at which polymorphisms were analyzed, only 19.6% of the alleles were shared between both population groups, while the remaining 42.5% and 37.8% were private alleles in the Ryukyu and mainland population groups, respectively (Table S9). Even in the mainland populations, which are regarded as new habitat regions, a relatively high proportion of private alleles was found, and there was no significant difference in frequency between the population groups (Wilcoxon signed rank test, *p* = 0.21).

**TABLE 2:** Genetic diversity indices (means ± SDs) of 53 polymorphic microsatellite DNA markers in *Melittobia sosui*.

Population^a^	Number of individuals analyzed	Number of alleles	Allelic richness	Gene diversity
Per population				
Iriomote	14	1.30 (± 0.64)	1.28 (± 0.59)	0.073 (± 0.173)
Ishigaki	25	1.34 (± 0.71)	1.29 (± 0.62)	0.064 (± 0.140)
Kanagawa	10	1.30 (± 0.50)	1.30 (± 0.50)	0.115 (± 0.200)
Per group^b^				
Ryukyu	42	1.72 (± 1.10)	1.49 (± 0.74)	0.086 (± 0.152)
Mainland	11	1.38 (± 0.53)	1.38 (± 0.53)	0.133 (± 0.205)

^a^ Only populations with sufficient sample sizes are represented.

^b^ The Ryukyu group consists of the Iriomote, Ishigaki, and Okinawa populations, and the mainland group consists of the Shizuoka and Kanagawa populations.

### 3.4 PCR Detection for *Wolbachia* Infection

An amplified product of the expected size for the *wsp* gene region was not detected in any of the samples examined, in contrast to the positive control of *M. australica* , suggesting that the sampled populations of *M. sosui* in this study were not infected by *Wolbachia*.

## 4 Discussion

We examined the population genetic structure and genetic diversity within and between native and invasive populations of *M. sosui* using mitochondrial and nuclear microsatellite DNA markers. Invasive populations are generally expected to exhibit reduced genetic diversity due to bottlenecks associated with founder events. As expected, the present study revealed that there was no variation in mitochondrial DNA in the invasive mainland populations, with only one haplotype that was included in the haplotype variation observed in the native Ryukyu populations. However, there were considerable variations in microsatellite DNA in the mainland populations, which were comparable with those in the Ryukyu populations. Moreover, in the mainland, 45.5% of the microsatellite loci contained private alleles that were not found in the Ryukyu populations. These results suggest that the mainland populations could have diverged from the Ryukyu populations, but the timing of the divergence was not recent as it takes time for new mutations to accumulate at the microsatellite loci.

One of the possible explanations for the high genetic diversity in microsatellite DNA in the invasive populations could be the introduction by multiple individuals. At the mitochondrial DNA level, all the individuals in the mainland populations shared a single haplotype, but it was not possible to distinguish whether the colonization originated from a single female, multiple females with the same haplotype, or multiple females with different haplotypes, with one becoming dominant owing to genetic drift or selection. However, regardless of the number of females at the origin of the mainland populations, the high genetic diversity in microsatellite DNA was caused by the high frequency of private alleles. Therefore, the high degree of divergence likely arose as novel mutations after the mainland populations diverged from the Ryukyu populations.

The contrast between the lack of mitochondrial variation and substantial microsatellite variation found in the mainland populations may be explained by the difference in mutation rates between mitochondrial and microsatellite DNA. In general, the mutation rate of microsatellites is higher than that of mitochondria. The mutation rate in the mitochondrial COI region was estimated to be 7.4 × 10^−5^ per generation in the parasitoid wasp *Nasonia* (Raychoudhury et al. 2010), which belongs to the same superfamily as *Melittobia*. The microsatellite mutation rate is typically on the order of 10^−3^–10^−5^ per locus per generation (Ellegren 2004; Chapuis et al. 2015) and falls within the same range in Hymenopteran species (Moritz et al. 2003; Schmid‐Hempel et al. 2007). Although the mutation rate may vary depending on the effective population size and population structure of the species, the proportion of microsatellite loci at which mutation did not occur during generations (*t*) can be calculated using the mutation rate per locus per generation (*μ*) as follows:

(1) $$1-P={\left(1-\mu \right)}^{t}$$

where, *P* is the proportion of loci with mutated alleles. Given that *μ* = 1.0 × 10^−3^ to 1.0 × 10^−5^ from the literature and *p* = 0.455 as obtained in the present study, the divergence time between the mainland and Ryukyu populations is estimated to be 6.1 × 10^2^–6.1 × 10^4^ generations ago. This result implies that the observed proportion of mutations at microsatellite loci could arise before one mutation occurs in the mitochondrial COI region. Moreover, although genetic diversity is generally expected to be lower in invasive populations than in native populations, the genetic diversity of microsatellite DNA in the mainland populations was similar to that in the Ryukyu populations. This result could be because the mutation rate of microsatellite DNA is so high that genetic diversity will converge at the same level for a long time (Nauta and Weissing 1996). Additionally, certain biological traits of the species, such as the number of generations per year, may also contribute to this convergence.

Along with the high mutation rate in microsatellite DNA, the parasitic life history of *Melittobia* species may facilitate the accumulation of newly emerged mutations. The parasitic lifestyle is expected to increase the nucleotide substitution rate due to small effective population sizes associated with frequent founder events (Page et al. 1998; Kaltenpoth et al. 2012). In the case of *Melittobia*, in which individuals mate within their natal broods and closely related females often lay eggs on the same host patches, inbreeding continues to occur unless more than one unrelated female disperses to the same host to lay eggs (Matthews et al. 2009; Abe and Pannebakker 2017; Abe et al. 2021). The present study showed that *M. sosui* experiences strong inbreeding, as supposed by the high values of *F*is. Populations with smaller effective population sizes experience faster rates of substitution by genetic drift (Ohta 1992). If a mutation occurs in a small inbreeding lineage, the mutation will be fixed with a higher probability than in a large non‐structured population. Different mutations are likely to be fixed in each inbreeding lineage, resulting in greater genetic diversity in the whole population. Similarly, population subdivision into genetically isolated lineages and subsequent admixture are expected to lead to increased genetic divergence (Frankham et al. 2002; Hartl and Clark 2007).

Another possibility for the reduced variation in mitochondrial but not in nuclear microsatellite DNA could be a cytoplasmic sweep by maternally coinherited *Wolbachia*. Raychoudhury et al. (2010) found that North American populations of the parasitoid wasp *Nasonia vitripennis* presented much less microsatellite variation than European populations but exhibited a similar level of microsatellite variation, and concluded that mitochondrial variation was likely decreased by *Wolbachia*‐induced sweeps. However, in contrast to the present study, the microsatellite data revealed shared polymorphisms and did not diverge between the two population groups, ruling out the possibility that microsatellite diversity was newly acquired after divergence. Although the present study revealed that *M. sosui* was not infected by *Wolbachia*, other maternally inherited bacteria or genetic elements could be the cause of the observed reduced variation in mitochondrial DNA. However, to date, there is no evidence suggesting that the reproduction of *M. sosui* is affected by genetic elements. All the broods collected in the present study and laboratory strains of *M. sosui* always included at least one male, ruling out parthenogenesis. *Melittobia* species typically show a female‐biased sex ratio for their own adaptive reproduction (Abe et al. 2003, 2021; Matthews et al. 2009), suggesting that male‐killing and feminization are unlikely to yield additional benefits. Hybridization between a mainland (Kanagawa) strain and a Ryukyu (Ishigaki) strain, which were derived from different mitochondrial clusters (Figure 2), produced viable offspring (Jun Abe unpublished data), implying that cytoplasmic incompatibility does not occur between these strains.

Based on the observational data reported in previous studies (Assem and Maeta 1980; Assem et al. 1982; Maeta 1985; Maeta et al. 1996) and recent data from the field, we expected that the population of *M. sosui* on the Japanese mainland was founded by an individual(s) that migrated from the Ryukyu area after the 1980s. However, although the present mitochondrial data supported this hypothesis, the microsatellite data contradicted it. According to Equation (1) using the upper limit of the microsatellite mutation rate (*μ* = 1.0 × 10^−3^) and the fact that *Melittobia* species produce approximately 6 generations per year on the Japanese mainland (Maeta 1978), the mainland populations of *M. sosui* were estimated to have diverged from the Ryukyu populations at least 100 years ago.

How did *M. sosui* invade and settle in the mainland? One possibility is that *M. sosui* had already invaded the mainland over 100 years ago and was distributed at low densities. If the density was low, it might not have been collected in previous studies. Even in small populations, *Melittobia* species can sustain the population through repeated inbreeding. Furthermore, as discussed above, the distribution of small populations at low densities and their subsequent mixture may have promoted an increase in genetic diversity throughout the population. Alternatively, there could be a ghost population that diverged from the Ryukyu populations more than 100 years ago. This possibility is proposed by the absence of previous collection data for *M. sosui* in the mainland and the high proportion of private alleles in the mainland found in the present study. The ghost population might have formed in regions where previous studies on *Melittobia* were not conducted vigorously. The distribution of the ghost population might have gradually expanded and reached Shizuoka and Kanagawa on the mainland without experiencing bottleneck events. In future studies, collecting samples from regions not investigated in the present study, including Taiwan and the Asian continent, as well as comparing the physiological characteristics, specifically in response to temperature, between the Ryukyu and mainland populations would be useful to assess the above possibilities.

In conclusion, the present study revealed reduced mitochondrial diversity and high microsatellite diversity in invasive Japanese mainland populations of *M. sosui*. The reduced mitochondrial diversity suggested that the mainland populations were founded by one or several individual(s) that migrated from the native area. Although the high microsatellite diversity may be explained by the introduction of multiple individuals, the high diversity observed in the present study resulted from the high proportion of private alleles, which instead suggested that the diversity was acquired after the population diverged from the native population. These findings suggest that the divergence of the mainland populations from the native populations did not occur recently, contrary to the initial prediction. More generally, the present results emphasize the usefulness of molecular techniques, which could lead to different conclusions compared with observational or historical data.

## Author Contributions

**Jun Abe:** conceptualization (lead), data curation (lead), formal analysis (lead), funding acquisition (lead), investigation (lead), methodology (equal), project administration (lead), software (lead), writing – original draft (lead), writing – review and editing (equal). **Jun‐ichi Takahashi:** conceptualization (supporting), data curation (supporting), formal analysis (equal), investigation (supporting), methodology (equal), software (equal), writing – original draft (supporting), writing – review and editing (equal). **Koji Tsuchida:** conceptualization (supporting), formal analysis (supporting), investigation (equal), methodology (equal), software (supporting), writing – review and editing (equal).

## Conflicts of Interest

The authors declare no conflicts of interest.

## Supporting information


Tables S1–S9.

## Data Availability

The sequence data of the microsatellite loci developed in this study were available in DDBJ/ENA/GenBank under the accession numbers LC848221–LC848279. The sequence data of the mitochondrial COI gene region were available in DDBJ/ENA/GenBank under the accession numbers LC84064–LC848116.
